# Supplementary material for: Evolution of the Antigenic Landscape in Children and Young Adults with COVID-19 and MIS-C
Source: Vaccines (Basel). 2024 Jun 7;12(6):638. doi: 10.3390/vaccines12060638 (PMC11209438; doi:10.3390/vaccines12060638)
Supplement: Supplementary file 1 [file vaccines-12-00638-s001.zip › vaccines-2950078-supplementary.pdf]

Table S1: SARS-CoV-2 variants mutations introduced in the spike plasmid for production of SARS-CoV-2 pseudovirions for analysis in PsVNA.

SARS-CoV-2 variant	Mutations constructed in the spike plasmids
Alpha (B.1.1.7)	H69-V70del, Y144del, N501Y, A570D, D614G, P681H, T716I, S982A, and D1118H
Delta (B.1.617.2)	T19R,G142D,E156del,F157del,R158G,L452R,T478K,D614G,P681R,D950N
Omicron (BA.2)	T19I, delL24, delP25, delP26, A27S, G142D, V213G, G339D, S371F, S373P, S375F, T376A, D405N, R408S, K417N, N440K, S477N, T478K, E484A, Q493R, Q498R, N501Y, Y505H, D614G, H655Y, N679K, P681H, N764K, D796Y, Q954H, N969K
Omicron (BA.2.75)	BA.2 spike mutations (T19I, delL24, delP25, delP26, A27S, G142D, V213G, G339D, S371F, S373P, S375F, T376A, D405N, R408S, K417N, N440K, S477N, T478K, E484A, Q493R, Q498R, N501Y, Y505H, D614G, H655Y, N679K, P681H, N764K, D796Y, Q954H, N969K) + K147E, W152R, F157L, I210V, G257S, D339H, G446S, N460K, Q493reversion
Omicron (BA.3)	A67V, del69-70, del142-144, Y145D, del211, L212I, G339D, S371F, S373P, S375F, D405N, K417N, N440K, G446S, S477N, T478K, E484A, Q493R, Q498R, N501Y, Y505H, D614G, H655Y, N679K, P681H, N764K, D796Y, Q954H, N969K
Omicron (BA.4/BA.5)	T19I, delL24, delP25, delP26, A27S, del69 70, G142D, V213G, G339D, S371F, S373P, S375F, T376A, D405N, R408S, K417N, N440K, L452R, S477N, T478K, E484A, F486V, Q498R, N501Y, Y505H, D614G, H655Y, N679K, P681H, N764K, D796Y, Q954H, N969K
Omicron (BQ.1)	BA.4/BA.5 spike mutations (T19I, delL24, delP25, delP26, A27S, del69 70, G142D, V213G, G339D, S371F, S373P, S375F, T376A, D405N, R408S, K417N, N440K, L452R, S477N, T478K, E484A, F486V, Q498R, N501Y, Y505H, D614G, H655Y, N679K, P681H, N764K, D796Y, Q954H, N969K) + K444T and N460K.
Omicron (BQ.1.1)	BQ.1 spike mutations (T19I, delL24, delP25, delP26, A27S, del69 70, G142D, V213G, G339D, S371F, S373P, S375F, T376A, D405N, R408S, K417N, N440K, L452R, S477N, T478K, E484A, F486V, Q498R, N501Y, Y505H, D614G, H655Y, N679K, P681H, N764K, D796Y, Q954H, N969K, K444T and N460K) + R346T.
Omicron (XBB.1)	BA.2 spike mutations (T19I, delL24, delP25, delP26, A27S, G142D, V213G, G252V, G339D, S371F, S373P, S375F, T376A, D405N, R408S, K417N, N440K, S477N, T478K, E484A, Q493R, Q498R, N501Y, Y505H, D614G, H655Y, N679K, P681H, N764K, D796Y, Q954H, N969K) + V83A, Del144, H146Q, Q183E, V213E, G339H, R346T, L368I, V445P, G446S, N460K, F486S, F490S, R493Q

Figure S1

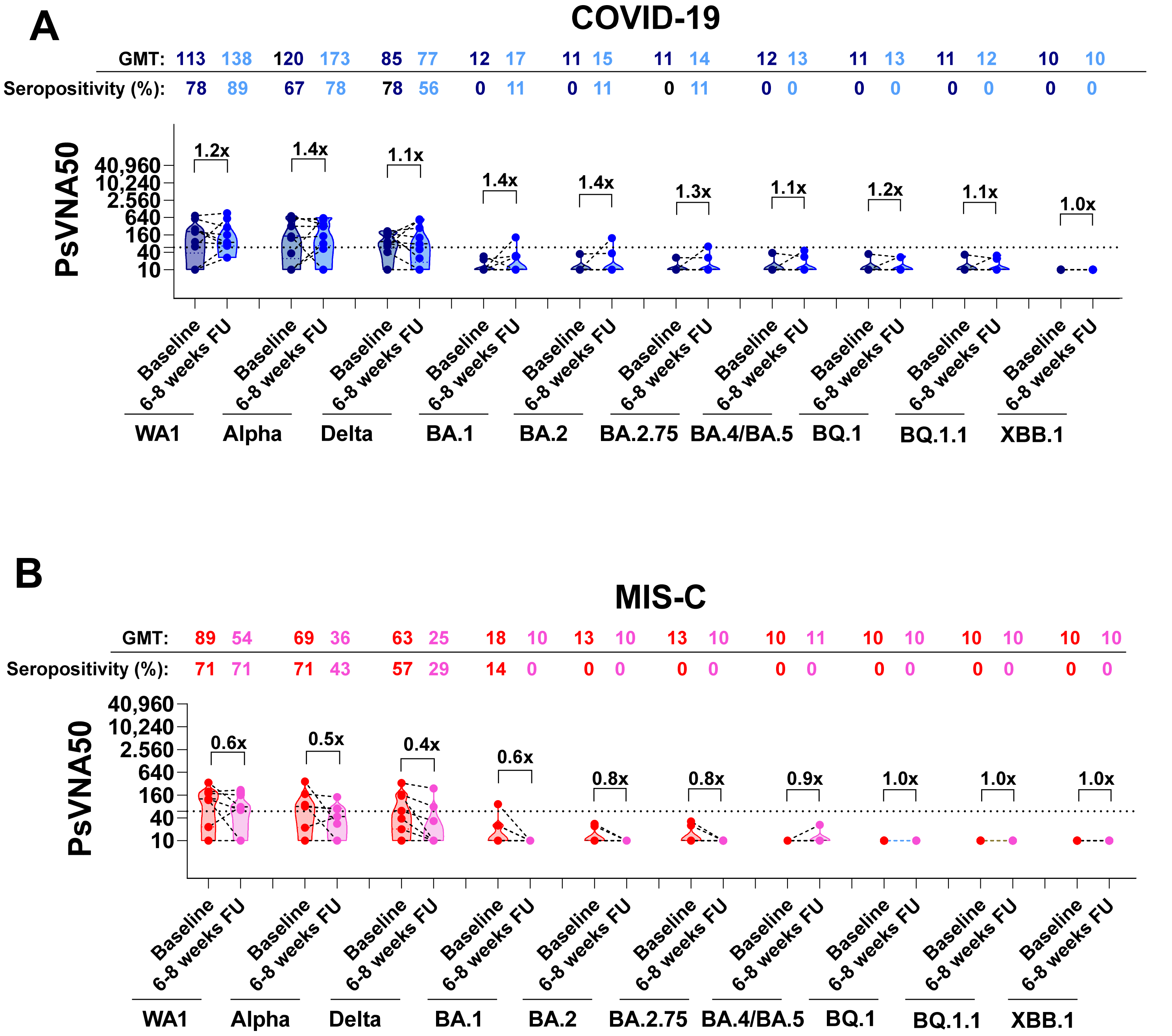

Figure S1: Evolution of neutralizing antibodies in children with acute COVID-19 or MIS-C against SARS-CoV-2 WA1 and Omicron subvariants overtime.
SARS-CoV-2 neutralization assays were performed using pseudoviruses expressing the spike protein of WA-1/2020 or the Omicron subvariants in 293-ACE2-TMPRSS2 cells. Paired samples at baseline and 6-8 weeks follow-up were evaluated for children with acute COVID-19 (n=11 in A) and MIS-C (n=9 in B). Geometric mean titer (GMT) values ± 95% CI of PsVNA50 (50% neutralization) titers for samples from either acute COVID-19 (baseline in dark blue and follow-up in light blue) or MIS-C (baseline in red and follow-up in pink) are shown. Fold change of 6-8 weeks follow-up compared with baseline samples is shown for PsVNA50 titers of each strain. All PsVNA experiments were performed twice and the researchers performing the assay were blinded to sample identity. The variations for duplicate runs was <7%. The data shown are average values of two experimental runs. Statistical differences were analyzed by Friedman test and no statistically significant differences were observed.

Figure S2

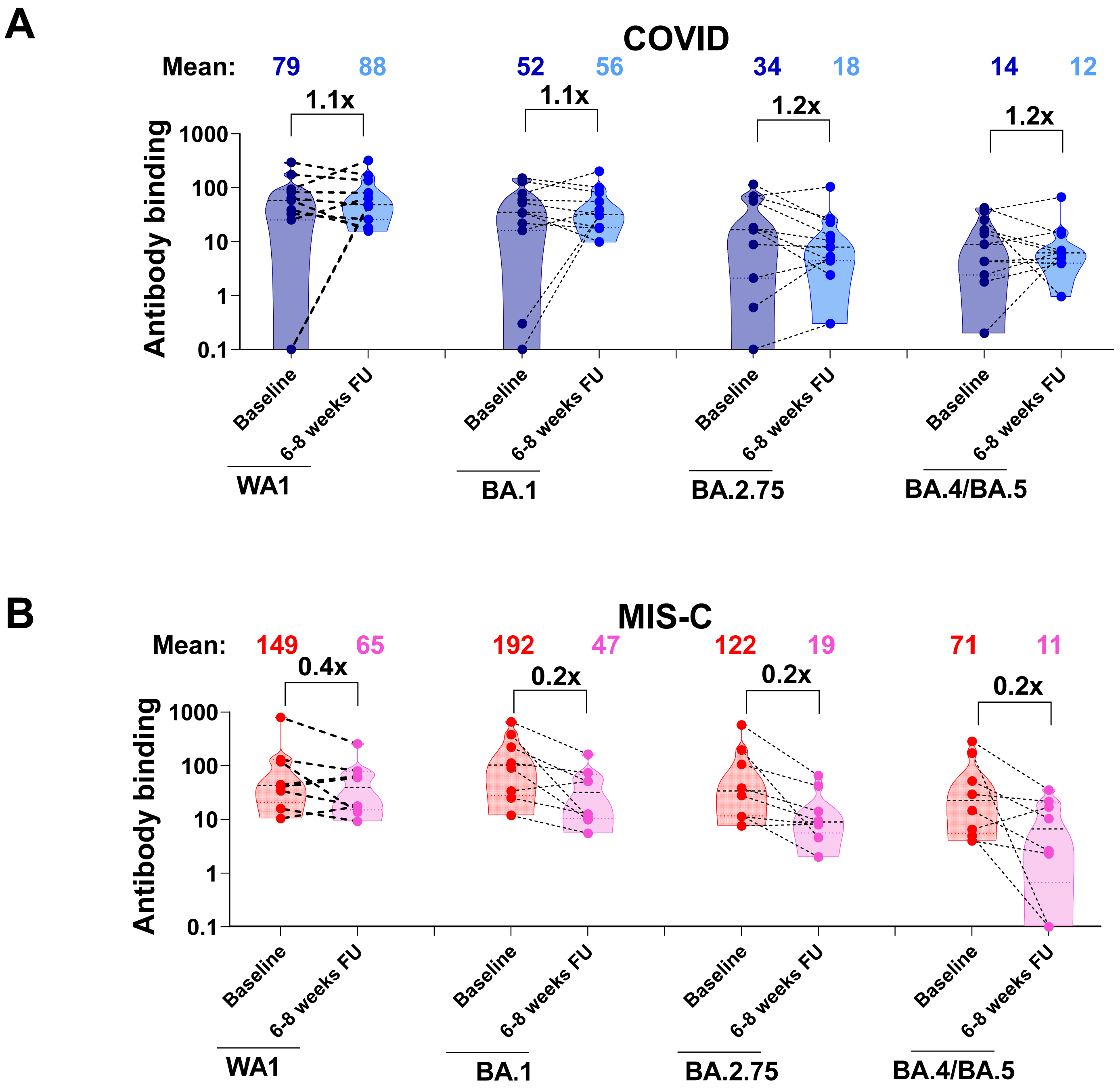

Figure S2. Binding antibodies in serum/plasma of children with COVID-19 vs MIS-C vs vaccination to the receptor binding domain of spike protein from ancestral SARS-CoV-2 WA1 strain and Omicron variants.
Total antibody binding (determined by maximum resonance units, Max RU) of 1:10 diluted serum or plasma to purified WA-1 RBD was measured by SPR. Data is shown for paired samples at baseline and 6-8 weeks follow-up for children with acute COVID-19 (n=11 in A) or MIS-C (n=9 in B). Mean antibody binding values are shown and are color coded by group. Fold change of 6-8 weeks follow-up compared with baseline samples is shown for RBD binding antibodies of each strain. All SPR experiments were performed in duplicate and the researchers performing the assay were blinded to sample identity. The variations for duplicate runs of SPR were <5%. The data shown are average values of two experimental runs. The statistical significances between the variants were performed using Friedman test. No statistically significant differences were observed.
